# New *Wolbachia pipientis* Genotype Increasing Heat Stress Resistance of *Drosophila melanogaster* Host Is Characterized by a Large Chromosomal Inversion

**DOI:** 10.3390/ijms232416212

**Published:** 2022-12-19

**Authors:** Aleksandra E. Korenskaia, Olga D. Shishkina, Alexandra I. Klimenko, Olga V. Andreenkova, Margarita A. Bobrovskikh, Natalja V. Shatskaya, Gennady V. Vasiliev, Nataly E. Gruntenko

**Affiliations:** 1 Institute of Cytology and Genetics SB RAS, 630090 Novosibirsk, Russia; 2 Kurchatov Genomics Center, Institute of Cytology and Genetics, SB RAS, 630090 Novosibirsk, Russia; 3 Department of Natural Sciences, Novosibirsk State University, Pirogova St. 1, 630090 Novosibirsk, Russia

**Keywords:** *Wolbachia*, wMelCS, wMelPlus, *Drosophila melanogaster*, genome assembly, genome comparative analysis, chromosomal inversion

## Abstract

The maternally transmitted endocellular bacteria *Wolbachia* is a well-known symbiont of insects, demonstrating both negative and positive effects on host fitness. The previously found *Wolbachia* strain wMelPlus is characterized by a positive effect on the stress-resistance of its host *Drosophila melanogaster*, under heat stress conditions. This investigation is dedicated to studying the genomic underpinnings of such an effect. We sequenced two closely related *Wolbachia* strains, wMelPlus and wMelCS^112^, assembled their complete genomes, and performed comparative genomic analysis engaging available *Wolbachia* genomes from the wMel and wMelCS groups. Despite the two strains under study sharing very close gene-composition, we discovered a large (>1/6 of total genome) chromosomal inversion in wMelPlus, spanning through the region that includes the area of the inversion earlier found in the wMel group of *Wolbachia* genotypes. A number of genes in unique inversion blocks of wMelPlus were identified that might be involved in the induction of a stress-resistant phenotype in the host. We hypothesize that such an inversion could rearrange established genetic regulatory-networks, causing the observed effects of such a complex fly phenotype as a modulation of heat stress resistance. Based on our findings, we propose that wMelPlus be distinguished as a separate genotype of the wMelCS group, named wMelCS3.

## 1. Introduction

Environment plays the largest role in creating unfavorable conditions for the survival of an organism. The most common causes of abiotic stress are unpredictable temperature fluctuations and other climate-dependent phenomena. In response to these fluctuations, physiological and behavioral changes occur in order to help living organism to adapt to the new conditions. These adaptive responses ensure the effective regulation of reproduction and ageing in most animals, including in an important model organism *Drosophila melanogaster* (Diptera: *Drosophilidae*).

On the other hand, an important factor of biotic nature affecting the biology of organisms is the potential endosymbiotic interference in many of their biochemical pathways. Bearing endosymbionts is usually rather costly to the host, and can lead to a number of trade-offs. To minimize some of the costs of living, some symbionts develop a mutually beneficial relationship. Microorganisms coexisting with their hosts can provide them with vitamins and essential amino acids [1,2]. In some cases, the presence of endosymbionts has a great impact on the host’s response to stress [3,4]. Thus, two major factors, the harsh environment and endosymbiotic presence could be linked through a shared impact on the stress-response.

One of the most widely spread endosymbionts of insects, spiders and nematodes is α-Proteobacteria *Wolbachia* [5,6]. An overwhelming amount of literature has been published on the subject of interaction between the host and *Wolbachia* (reviewed in [7]). Yet, the symbiosis of insects and *Wolbachia* remains a topic shrouded in mystery.

Firstly, the relationships between *Wolbachia* and its host have both mutualistic and parasitic traits, which complicates our understanding of the interactions within this system (reviewed in [7,8,9]). To make an accurate assessment of *Wolbachia’s* influence on the host it is necessary to include data on as many associations between strains of this bacterium and species (or even lines of the same species) of host-insects, as feasible. This task seems almost impossible, since new strains with new traits are discovered nearly every year. The numerous observed effects caused by the symbiont on the different aspects of life of the hosts suggest that determining whether *Wolbachia* is friend or foe to the host is not as simple as separating black from white. Researchers focus instead on the specific effects of *Wolbachia* on the objects of their interest. This approach allows us to make accurate judgments on a case-by-case basis. The two most-known deleterious effects *Wolbachia* causes are cytoplasmic incompatibility (CI), present in many species [10] and severe lifespan shortening, caused by the infamous wMelPop strain first found in the laboratory line of *Drosophila melanogaster* [11]. CI effects *Wolbachia* transmission, and majorly increases the infection frequencies in the host-populations. In this case, the *Wolbachia*-carrying hosts reproduce better than the hosts without *Wolbachia*. Meanwhile, wMelPop effects the host viability in a negative way, putting the host at a disadvantage. Despite the fact that the success of the symbiont directly depends on the success of the host, *Wolbachia* does not always contribute positively to the host’s competitive ability. Of course, many strains of *Wolbachia* are seemingly neutral: they are not harmful and nor do they provide any registered benefits to their host.

Secondly, most researchers put the spotlight on the manipulations of the host species’ reproduction, and not on a possible influence of *Wolbachia* on the processes in the somatic cells. This has resulted in the limited attention that this area of research has received in the literature. For cases where the alteration of the host reproduction cannot explain the prevalence of *Wolbachia* in the host population, endosymbiont effects on host fitness can be an alternative reasonable explanation [12]. For example, it was shown that the resistance of *Drosophila melanogaster* to RNA viruses depends on the presence of *Wolbachia* [13,14]. A combination of the host’s genetic background and the *Wolbachia* strain plays a role in changing behavioral traits such as host locomotor-activity [15,16,17] and rate of mating [16,17,18]. Identical wMelCS variants have effects of different intensity on *D. melanogaster* stress-resistance, depending on the host line [19]. A similar idea is expressed by Dean [20]. Therefore, nature and the magnitude of different effects on host fitness also vary, depending on the combination of the host line and the strain of *Wolbachia*. 

These examples show us that the relationship between small taxonomic units, such as lines and strains, are more complex than one might expect, and these nuances need to be carefully considered. This leads us to the third big problem of *Wolbachia* studies: small taxonomic units within the same species are often indistinguishable by scientists, even though this distinction could be key in the matter of research. *Wolbachia* infection in *D. melanogaster* was first described as a single clonal infection, and for this reason it is still often referred to as a single *Wolbachia* strain—wMel. However, Riegler et al. in [21] separated five different genotypes of *Wolbachia* of *D. melanogaster*, based on polymorphic markers. It was theorized that several distinct lineages originated from a single ancestral *Wolbachia* infection [21,22], although the exact number of lineages is yet to be specified. Newly found variants of infection in *D. melanogaster* are usually referred to in the literature as strains, despite the fact that this title is also reserved for all *Wolbachia* infections in this host-species. Finding a new strain is a great stroke of luck for the researcher. However, often no information is provided in a study introducing this strain to fit it into one of the distinct genotypes. The robust tool for genotyping usually serving this purpose is a system called multilocus sequence typing (MLST).

When it comes to naming a novel strain, there are many approaches: the name might represent a certain mutation in a known locus, a certain phenotype of the host, or the geographical origin of a new strain [23,24]. As pointed out by Iturbe-Ormaetxe et al. [25], *Wolbachia* strains with identical MLST profiles can have different names to indicate the geographical area from where the strain was isolated. Unfortunately, this creates confusion and a necessity to reach out for clarification. Thus, similar names sometimes convey different information, and can be easily mixed up.

We had previously found a novel strain of *Wolbachia pipientis* named wMelPlus, which provides an increase in resistance to the heat stress of its host, the model species *D. melanogaster* [4]. Such increase in fitness is a serious advantage, yet its mechanisms are still unknown. The discovery of this novel strain can provide a promising source for studying often overlooked, hidden *Wolbachia* diversity. According to Ilinsky [22], this strain obtained from the wild-type D. melanogaster line w153 is a variant of the wMelCS genotype, as well as the pathogenic wMelPop. 

Genomic analysis might be at the heart of our understanding of the mechanisms by which endosymbionts might provide fitness benefits to the host. The accessibility of the complete genomes of both species in symbiosis creates an ideal system for studying these effects. The past fifty years have seen increasingly rapid advances in the field of deciphering nucleotide sequences. Next-generation technologies have become key instruments in genomics. 

Using the comparative genomic analysis, the pathogenic effect of wMelPop was shown to correlate with the copy number of the Octomom sequence [26]. This method proved to be reliable for revealing the nature of such a vital effect on fitness. 

Here, we sequence, assemble and compare genomes of two closely related *Wolbachia* strains of the wMelCS genotype: wMelCS^112^ and wMelPlus. The main purpose was to find the differences between the *Wolbachia* strain which has a positive effect on *D. melanogaster*’s stress resistance and the *Wolbachia* strain which does not.

Rare *Wolbachia* variants of *D. melanogaster* are often found in laboratory stocks. Did they occur in natural populations first, or did they arise under laboratory conditions?

This project provides an important opportunity to advance our understanding of the complex relationships between *Wolbachia* and its insect hosts. It is hoped that this study will lead to new insights into the evolution of *Wolbachia* infections.

## 2. Results and Discussion

### 2.1. Hybrid-Assembling Genome of a Novel Wolbachia Strain

We have assembled the genomes of two *Wolbachia* strains (wMelCS^112^ and wMelPlus), based on sequencing the material obtained from the ovaries and homogenized, whole-female infected flies of *Drosophila melanogaster* Bi90^wMelCS112^ and Bi90^wMelPlus^ lines (see Section 3, for details). The combination of two types of sequencing technologies, the Oxford Nanopore^®^ (Oxford Nanopore Technologies, Oxford, UK), yielding long-read libraries with the Illumina MiSeq^®^ (Illumina, San Diego, CA, USA), allowed us to assembly a high quality chromosome-level assembly of the *Wolbachia* genome. The quality of the resulting assemblies, including the results of genome polishing, have been assessed using contiguity and orthology measures, and the comparison is presented in Table 1.

### 2.2. From Assembly to Annotation of Genomes

We compared the assembled genomes of the most closely related yet differing in their effect, *Wolbachia* strains—wMelPlus and wMelCS^112^, to identify the differences in gene-set composition and their polymorphisms. The annotation of the obtained assemblies showed that there are minor differences between the analyzed strains.

We identified variation in gene set between wMelPlus and wMelCS^112^, using OrthoFinder [27]. Only two groups of orthologous genes (orthogroups), yielding rather small and uncharacterized protein products, revealed discrepancies between the two analyzed strains (see Table 2).

The gene of one of these proteins (WMELCS112_00485) turned out to be unique for a wMelCS strain. Its gene lies in the same operon as another uncharacterized gene (WMELCS112_00486), which contains the TrbC pilin domain and is an orthologue of WMELPLUS_00417. Thus, it appears that wMelPlus has lost one of the genes from this operon, with unknown function.

Another orthogroup is represented by three gene copies in wMelCS^112^ (WMELCS112_00749, WMELCS112_00771, WMELCS112_00896) and two in wMelPlus (WMELPLUS_00748, WMELPLUS_00770). These genes are single (not included in any known operon), uncharacterized, and do not contain any known domains.

Furthermore, after SNP calling we identified 7 SNPs residing in coding sequences, including synonymous and missense variants (see Table 3).

Most of the identified SNPs cannot be attributed to any known genes involved in the host–symbiont interaction. Thus, DNA gyrase subunit B is known to be involved in maintaining the bacterial chromosome in an underwound state [28]; the IS4-family transposase is required for excising and inserting the corresponding mobile element [29], and the tyrosine recombinase XerD is known to participate in a site-specific recombination in prokaryotes, serving to resolve dimers of circular chromosomes [30]. Therefore, we conclude that the stress-resistant phenotype might not be determined by genetic polymorphisms, at this level.

### 2.3. Large Chromosomal Inversion as a Probable Cause for a Phenotype of Interest

Besides these minor differences in the gene-set composition and SNPs, we found a large inversion in the region (308,915…531,369) spanning 222,454 bp in the wMelPlus strain. To rule out the possibility of an artifact, we mapped long reads on the reference genome and visualized the results with an IGV genome-browser. The regions encompassing the boundaries of the inversion are highly covered with the long reads. Moreover, the boundaries of the inversion coincide with the gene WMELPLUS_00339 IS5 of family transposase ISWpi1 on one side, and with the gene WMELPLUS_00563 IS5 family transposase ISWpi1 on another side. There are numerous studies showing that various transposases, including the IS5 family, are able to promote the emergence of inversions, due to homologous recombination between the transposase genes [31,32,33]. These facts indicate that it is a true inversion and not a misassembly. It is known from the literature that such a rearrangement can affect the expression of respective genes and change a phenotype, even without mutations in ORFs [34]. For instance, a 2.1 Mb inversion for the *E. coli* strain resulting in an increase in resistance to antibiotics and sodium, has been shown [35].

This inversion contains 102 genes with a known annotated product, and 207 genes in total (see Tables S1 and S4 in Supplementary Materials). It constitutes ~1/6 of *Wolbachia* genes, including those concerned with basic cellular processes, enzymes and transporters. Furthermore, there are also a large number of hypothetical proteins of unknown function, which can be investigated in future studies. Unlike such microorganisms as *Lactococcus lactis* or *Mycoplasma genitalium* and *Mycoplasma pneumonia*, which are known for ~80% of their genes being located on the leading strand [31,36], *W. pipientis* shows no clear gene-strand bias (we discuss this point in detail in Section 2.4) while producing viable phenotypes under large inversions.

**Figure 1:**
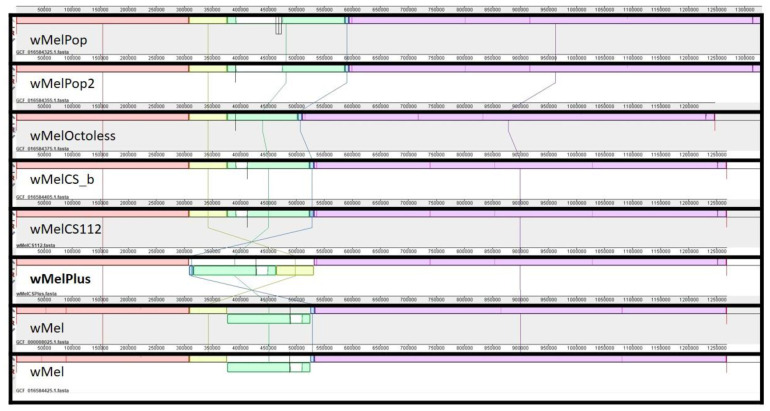
The inversion map resulting from the comparative genomic analysis of wMel and wMelCS groups of *Wolbachia pipientis* strains. Different colors represent homologous sequence blocks. Discontinuous joints of blocks depict the inversion breakpoints. White blocks represent Octomom, which is absent in the wMelOctoless strain [37]. These are the coordinates of the homologous sequence blocks in the wMelPlus genome: (1…308,920)—red, (308,921…316,275)—blue, (316,276…463922)—green, (463,923…531,364)—yellow.

We hypothesize that dysregulation of the involved genes has interfered in the crosstalk between *Drosophila*-*Wolbachia* genetic regulatory circuits, causing observed effects in such a complex phenomenon as fly heat stress response. One of the well-known regulatory complexes is Octomom—a region containing eight *Wolbachia* genes, whose loss or amplification is responsible for wMelPop over-proliferation [26,37]. Therefore we examined the position of Octomom in regard to the identified inversion. Only one copy of Octomom is present in the wMelPlus genome, just as in the wMelCS_b genome. However, in wMelPlus, Octomom is located inside the inversion on the lagging strand (see Figure 1), which could have an impact on *Wolbachia* proliferation in a host; however, there is no clear evidence yet.

### 2.4. Comparative Genomic Analysis of wMel and CS Groups of Wolbachia Pipientis Strains

To shed light on the issue of inversions in *Wolbachia* symbionts of *D. melanogaster,* we performed a comparative genomic analysis of those wMel and wMelCS groups of *Wolbachia pipientis* strains whose complete genomes were available (for details see Section 3.4). The results of whole genome comparisons are presented in Figure 1.

It is known that the inversion between wMel and wMelCS is a widely used trait for *Wolbachia* genotyping, to distinguish these strains using the MLST approach [21]. It is even more intriguing that the inversion in a similar region occurred in wMelPlus independently, since other MLST markers indicate that this strain definitely belongs to the wMelCS group. However, our analysis showed that there are some differences between these similar inversions in wMelPlus and wMel, namely, “blue” (308,921…316,275) and “yellow” (463,923…531,364) regions (see Figure 1), and we will regard these as unique inversion blocks in future. It is worth noting that since the wMelPlus inversion is not the same inversion as we see in the wMel strain, the product for wMelPlus is not synthesized using the primers for the inversion identification proposed by Riegler et al. [21], thereby classifying wMelPlus as a member of the CS group.

There are genes located in these particular regions (see Tables S2 and S3) that are of specific interest because they might be involved in the induction of a stress-resistant phenotype, which manifests in *D. melanogaster* infected with wMelPlus but not with other known *Wolbachia* genotypes *D. melanogaster* endosymbionts [4].

When speaking of potential bacteria–host interaction, it is important to draw attention to those genes encoding products that excrete into the environment of the bacterial cell, and thereby are able to directly interact with the host cell. Among the list of genes of sensing, secretion and transcription-regulation [38] that might be involved into the host–microbe interactions of *Wolbachia*, there is only one protein-encoding gene from the unique inversion blocks which has a product that contains signal peptides, indicating its putative extracellular localization—WMELPLUS_00535 (“yellow” region)—presumably a trans-membrane protein containing type IV-secretion-system domain (TrbC/VIRB2 pilin), which has changed its location due to inversion from lagging to leading strand. There are also three copies of genes coding WsnRNA46 (“yellow” region)—a small non-coding RNA, which is known for being excreted into the host cells and employing the mechanism of RNA-interference to up-regulate the Dynein heavy chain gene (*Dhc*)—a microtubular motor protein, which is important for *Wolbachia* transmission into *Drosophila* oocytes [39,40].

As for genes located near the inversion, the position of gene SecA (WMELPLUS_00566) is less than two Kbases from the distal (right) edge of inversion. The SecA gene is part of the SEC system, which translocates proteins across or into the inner membrane, and can also be involved in the regulation of various processes mediating host–bacteria interactions.

Following the hypothesis that dysregulation of the involved genes could interfere with the established genetic regulatory circuits, due to the inversion, we focused our attention on some co-regulated groups of *Wolbachia* genes known from the literature. We examined three mutually non-exclusive classes of co-expressed genes discussed in [41]:

1. GroES/WD0308, DnaK/WD0928, Hsp90/WD1277, GroEL/WD0307: genes that show high relative expression in *Drosophila* embryos, with down-regulation later in the life cycle.
2. WspB/WD0009, TerC/WD0194, SPFH domain/WD0482, type II secretion/WD0500, HlyD/WD0649, type I secretion/WD0770, VirB3/WD0859, Rhoptry surface protein related/WD1041, WD0191, WD0385, WD0438, WD1213, DksA/WD1094: the up-regulated genes that increase in relative expression starting with the early larval stages and carrying on into adulthood, with decreases at the late larval (12 hr) stage and increases at the white prepupal (2 and 3 d) stages.
3. WD0291, WD0292, WD0438: genes that show up-regulation primarily in *D. melanogaster* adults, with higher expression in adult males relative to adult females, at the same age.

The same gene identifiers as in [41] were taken, from *Wolbachia* wMel reference-genome locus tags (Ensembl Genomes Release 24, *Wolbachia*_endosymbiont_of_*Drosophila*_melanogaster.GCA_000008025.1.24). We found that only several genes from the second (WD0482/WMELPLUS_00427, WD0500/WMELPLUS_00443, WD0385/WMELPLUS_00501, WD0438/WMELPLUS_00383) and third classes (WD0438/WMELPLUS_00383) are located in the identified inversion, and only one of them (WD0385/WMELPLUS_00501) is located in a unique inversion block (namely, “yellow” region) and therefore changes its localization compared with both the wMel and wMelCS strains, whereas other proteins from the second class stay outside the inversion. The corresponding protein contains the ankyrin repeat domain, which might indicate its potential role in protein–protein interactions.

**Figure 2:**
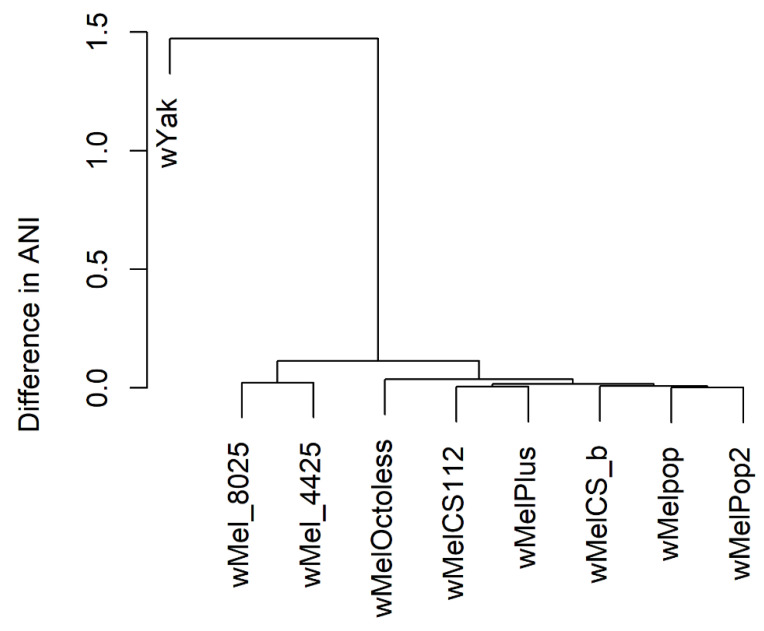
The dendrogram reflecting genome similarity between the analyzed strains based on average nucleotide-identity (ANI) values. wMel_8025 and wMel_4425 correspond to the wMel strain genome assemblies GCF_000008025.1 and GCF_016584425.1, respectively.

Despite the fact that the genomes of *Wolbachia* endosymbionts of *Drosophila melanogaster* are relatively similar, carrying a small number of polymorphisms, the classification of these strains using average nucleotide identity (ANI), demonstrates meaningful topology (see Figure 2).

The wMel and CS groups form two clusters, and the wMelPop and wMelPop2 strains also group together. These results give us some evidence that the closest relative of wMelPlus is the wMelCS^112^ strain.

As has been mentioned already, many bacteria tend to demonstrate preference for gene allocation on the leading rather than the lagging strand, because the latter suffers from replisome and RNA-polymerase collisions [42], which decrease the replication rate and affect bacterial fitness [43,44]. The effect of such a preference is known as gene-strand bias (GSB) [34]. We calculated GSB_p_ (see formula (2)) for the *Wolbachia* strains that we used for the comparative genomic analysis, and the results are presented in Table 4.

The results of gene-strand bias analysis show that the analyzed *Wolbachia* strains demonstrate no clear strand preference; however, bearing in mind the general trend among bacteria for GSB to be greater than 50% [45,46] one can assume that the inversions in the wMel and wMelPlus strains, which result in a decrease in GSB_p_ values below this threshold, are of recent origin, and wMelCS is more ancient than both the wMel and wMelPlus strains, which is in accordance with the replacement hypothesis [21].

Endosymbiosis of *Wolbachia* and *Drosophila* puts a number of constraints on the evolution of bacterial species, to ensure a high-tuned coupled functioning of essential processes. Bacteria, having relatively compact genomes, cannot afford drastic changes in their protein repertoire and have to resort to more subtle regulation such as chromosomal rearrangements, in particular, inversions. Inversions are known to have a large impact on different aspects of an organism’s life—from suppressing recombination between co-adapted genes to interfering with the regulatory networks influencing gene expression [47], which can strongly affect the phenotype [48,49] and adaptation to an environment [50,51]. Although their effects are more pronounced in eukaryotes, inversions are widespread in prokaryotes, too [52,53,54], altering the expression of genes [48] and even bringing about diversity at a cell-population level [49]. We suggest that the inversion that we discovered by comparing two closely related *Wolbachia* strains—wMelPlus and wMelCS^112^—could disrupt the established groups of genes and regulatory elements (or lead to the assemblage of new groups such as these), which might influence the phenotype under heat stress conditions. We have identified a number of genes in unique inversion blocks of wMelPlus, some of them known to be members of already known co-expressed classes, which might be involved in the induction of a stress-resistant phenotype in the host, and are worth investigating in future studies. Thus, as a result of our comparative genomic analysis, we put forward the hypothesis that the discovered chromosome inversion in the wMelPlus *Wolbachia* strain is the probable cause of the corresponding *Drosophila*’s stress-resistant phenotype, and we suggest that this hypothesis requires further experimentation. It is important to note that a slightly smaller inversion [21] in this region in the wMel strain has been previously described; thus, the emergence of such an “inversion hotspot” might indicate that there is a selection favoring this type of chromosomal rearrangement in *Wolbachia*. While detailed phylogeny of *Drosophila* endosymbiotic *Wolbachia* remains obscure, this study sheds some light on its particular features and the role of inversions in their diversity.

## 3. Materials and Methods

### 3.1. Drosophila Lines and Rearing

The females of the *D. melanogaster* wild-type line Bi90 carrying either wMelCS^112^ or wMelPlus *Wolbachia* strains were taken for the study at the age of 11 days. Before that, flies were maintained on standard food (agar-agar, 7 g/L; corn grits, 50 g/L; dry yeast, 18 g/L; sugar, 40 g/L) in the MIR-554 incubator (Sanyo, Osaka, Japan) at 25 °C under a 12:12 h light–dark cycle.

### 3.2. Genomic DNA Extraction and Sequencing

*Wolbachia* DNA was extracted from a whole *Drosophila* female (150 flies for one sample) or its ovaries (150 pairs for one sample), following the protocol described in [55].

For MiSeq sequencing, 1 mkg DNA from whole flies was fragmented using a Covaris M220 sonicator with parameters optimized for a maximum fragment-size of approximately 400 bp. Barcoded-genome libraries were prepared, using 50 ng of fragmented DNA, with Roche KAPA Hyper Prep Kit, KAPA UDI adapters, according to the manufacturer’s protocol for dual size-selection. The amplification of libraries was carried out in 9 cycles. The quality and molarity of the libraries were determined using a Bioanalyzer BA2100 and Qubit fluorim-eter. After normalization, the barcoded libraries were pooled and sequenced, using the MiSeq Reagent Kit v2 (500-cycles).

For Nanopore sequencing, 1 mkg DNA was fragmented by pipetting five times in 20 mkl volume. Libraries were prepared using the NEBNext^®^ Companion Module for Oxford Nanopore Technologies^®^ Ligation Sequencing (Oxford Nanopore Technologies, Oxford, UK), according to the manufacturer’s protocol without barcoding, and using a long-fragment buffer at the washing step. Nanopore sequencing was performed using the MinION Mk1C device, SpotON Flow Cell (R9.4) and Ligation Sequencing Kit (Oxford Nanopore Technologies, Oxford, UK).

### 3.3. Genome Assembly, Polishing and Annotation Pipeline

First, standard quality and contamination control procedures were performed, using fastp [56] and BWA against the probable contaminants such as the host’s nuclear and mitochondrial sequences, and the human and synthetic sequences (vector contamination, adapters, linkers, and primers from the UniVec database).

**Figure 3:**
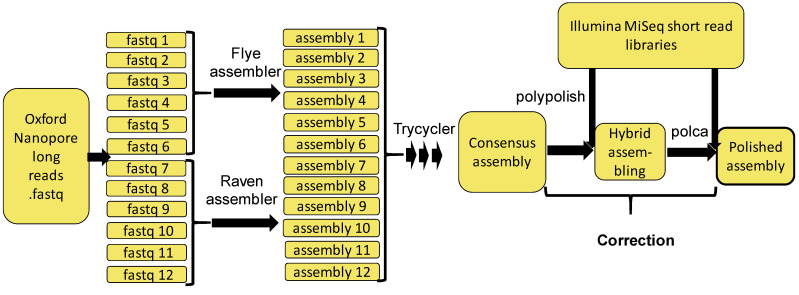
The bioinformatic genome assembly, correction and polishing pipeline used in the current work. Solid lines illustrate the work- and data-flow.

We used the Trycycler [57] assembler for a long-read assembling of *Wolbachia* genomes based on Oxford Nanopore sequencing data (the assembly and polishing pipeline is presented in Figure 3). The entire long-read library was split into 12 subsets and two parts—the first six were assembled with Flye [58] and the remaining six subsets with Raven [59], before scaffolding with Trycycler. The consensus assembly generated by Trycyler was corrected during subsequent hybrid assembling with Illumina Miseq short reads, using Polypolish [60]. Genome polishing was performed using the POLCA [61] tool. We assessed the quality of the resulting assemblies using QUAST [62] and BUSCO [63].

The obtained genomes were annotated using the Prokka [64] annotation pipeline (v. 1.14.6), and additionally annotated with BLAST alignment against the CDD database. To collect more information about the genes located in the inversion, the operons for both strains were predicted using the Operon-mapper web service [65]. The localization of the products of the analyzed genes was predicted using the DeepTMHMM [66] and SignalP [67] web services. The domains of the poorly annotated genes were predicted by running the HMMER [68] against the Pfam database [69].

We also performed SNP calling in the coding sequences in the obtained assemblies based on Illumina Miseq short-read libraries. These reads, obtained from the wMelPlus strain, were mapped to the assembled genome of wMelCS^112^ using Snippy on the Galaxy platform [70]. The obtained variants were annotated using SnpEff [71] on the Galaxy platform with the upstream/downstream length equal to 0 bp, using the wMelCS^112^ genome as a reference. The comparisons of the gene sets for the wMelCS^112^, wMelPlus and the set of the reference genomes were made using OrthoFinder [27].

### 3.4. Comparative Genome Analysis

For the comparative genome analysis, we used the obtained assemblies for the wMelPlus and wMelCS strains and 12 reference genomes of *Wolbachia* endosymbionts of *D. melanogaster*, which were available on public databases (see Table 5). All assemblies were annotated using Prokka version 1.14.6.

These genomes were analyzed with a tool for phylogenetic orthology inference, OrthoFinder [27], which classified genes of the assemblies under analysis into orthogroups. These orthogroups were examined for the differences in number of genes, i.e., variations in orthogroup presence in a genome, including copy-number variation. The results were verified with BLAST.

The resulting chromosome-level annotated assemblies were compared with the already published genomes of *W. pipientis*. The multiple genome alignment of the wMelPlus, wMelCS^112^, and wMelCS_b strains was carried out using the ProgressiveMauve [76]. The mapping of the long reads onto the assembled genomes was performed using the IGV. The annotation was visualized with the Unipro UGENE [77].

The phylogenetic tree was reconstructed based on average-nucleotide-identity (ANI) values obtained with the fastANI tool [78]. The pairwise ANI-values were calculated between a number of *Wolbachia* endosymbionts of *Drosophila melanogaster* strains, using the complete-genome assembly level (see Table 5), the wMelCS^112^ strain, the wMelPlus strain, and wYak (GCF_018467115.1), which is the *Wolbachia* endosymbiont of *Drosophila yakuba*, thus representing an outgroup. The obtained matrix of ANI values was visualized as a dendrogram using the gplots package in R.

Given a genome with annotated genes, genome-strand bias (*GSB*) can be calculated using the following formula:

(1) $$GSB=\frac{\left|G^{+}\right|}{\left|G\right|}$$

where *G*^+^ is the set of genes located on the leading strand, and *G* is the total set of genes. Alternatively, similar characteristics can be calculated using a subset of protein-coding genes:

(2) $$GSB_{p}=\frac{\left|G_{p}^{+}\right|}{\left|G_{p}\right|}$$

where *G*^+^ is the set of protein-coding genes located on the leading strand, and *G* is the total set of protein-coding genes.

## 4. Conclusions

The genome assembly of a new *W. pipientis* strain was obtained. It exhibits enough quality for subsequent comparative-genomics analysis, and can be regarded as a promising source for studying different aspects of its endosymbiosis with *Drosophila melanogaster*.

The strain wMelPlus is shown to have such striking differences when compared with the known samples of wMelCS *Wolbachia* genomes, that it deserves to be defined as a separate genotype of the wMelCS group, named wMelCS3.

## Figures and Tables

**Table 1:** Genome statistics of the resulting genomes obtained from hybrid assembling for both *Wolbachia* strains and a reference genome. BUSCO analysis has been performed using the Rickettsiales_odb10 data set.

Genome Statistics	wMelPlus_TryCycler	wMelPlus_Polypolish	wMelPlus_Final	wMelCS_TryCycler	wMelCS_Polypolish	wMelCS_Final	wMelCS_ b (Reference)
Total length	1,266,704	1,267,811	1,267,850	1,266,679	1,267,816	1,267,849	1,267,843
GC content (%)	35.26	35.23	35.23	35.26	35.23	35.23	35.23
Duplication ratio	0.999	1	1	0.999	1	1	N/A
misassemblies	2	2	2	0	0	0	0
mismatches per 100 kbp	0.95	0.47	0.95	0.63	0.39	0.39	N/A
indels per 100 kbp	89.92	4.1	1.03	92.2	3.71	1.1	0
Complete and single-copy BUSCOs	232	361	361	234	361	361	361
Complete and duplicated BUSCOs	0	1	1	0	1	1	1
Fragmented BUSCOs	80	0	0	82	0	0	0
Missing BUSCOs	52	2	2	48	2	2	2

The polymorphisms between the two strains are discussed in Section 2.2, and we elaborate on the two misassemblies in wMelPlus compared with the reference wMelCS_b genome, in Section 2.3.

**Table 2:** The differences in the number of genes in orthogroups between the wMePlus and wMelCS^112^ *Wolbachia* strains.

Number of Genes in the Orthogroup wMelCS^112^	Number of Genes in the Orthogroup wMelPlus	Locus_tags in the wMelCS^112^ Genome	Product	Length (Aminoacid Residues)
1	0	WMELCS112_00485	Hypothetical	39
3	2	WMELCS112_00749, WMELCS112_00771, WMELCS112_00896	Hypothetical	58

**Table 3:** The SNPs between the wMePlus and wMelCS^112^ *Wolbachia* strains.

Position	Impact	Gene	Gene Locus_tag (wMelCS112/wMelPlus)	Product	Mutation	Substitution Type
104,567	Synonymous variant	gyrB	WMELCS112_00114/ WMELPLUS_00114	DNA gyrase subunit B	Ala465Ala	
108,911	Stop gained		WMELCS112_00118/ WMELPLUS_00118	IS4 family transposase	Glu443 *	Nonsense change
256,666	Missense variant		WMELCS112_00283/ WMELPLUS_00283	Hypothetical protein	Asn36Asp	Neutral AA is changed to acidic AA
396,812	Missense variant		WMELCS112_00444 /WMELPLUS_00458	Hypothetical protein	Glu616Lys	Acidic AA is changed to basic AA
624,101	Missense variant		WMELCS112_0065/ WMELPLUS_00654	Hypothetical protein	Ala619Val	Same class/polarity/charge substitution
726,291	Stop loss and splice-region variant	xerD_1	WMELCS112_00784/ WMELPLUS_00783	Tyrozine recombinase XerD	Ter310Glu	Gene extension
1,212,940	Synonymous variant		WMELCS112_01313/ WMELPLUS_01311		Gly491Gly	

* indicates a stop codon.

**Table 4:** GSB_p_ and the number of protein-coding CDS on the leading and lagging strands for *W. pipientis* strains.

Strain	Leading Strand (+)	Lagging Strand (−)	Total Number of CDS	GSB_p_ (%)
wMelPop	684	620	1304	52.45399
wMelPop2	684	620	1304	52.45399
wMelPlus	619	646	1265	48.93281
wMelCS112	652	615	1267	51.46014
wMelCS_b	650	618	1268	51.26183
wMelOctoless	638	611	1249	51.08086
wMel (GCF_000008025.1)	633	638	1271	49.8033
wMel (GCF_016584425.1)	630	639	1269	49.64539

The data of strain of the interest (wMelPlus) are highlighted in gray.

**Table 5:** The list of species under study (species for which proteomic data were collected) and corresponding assembly accessions.

Accession	Isolate/Strain	Assembly Level	Group	Phenotype
GCF_000008025.1	wMel	Complete Genome	wMel	[72]
GCF_000475015.1	wMelPop	Scaffold	wMelCS	It over-replicates, which causes severe life-shortening of its host [73]
GCF_014354335.1	wMelCS	Scaffold	wMelCS	[74]
GCF_014354345.1	wMel	Scaffold	wMel	[74]
GCF_016584325.1	wMelpop	Complete Genome	wMelCS	Causes early death of the host (carries additional copies of Octomom region) [37]
GCF_016584355.1	wMelPop2	Complete Genome	wMelCS	Causes early death of the host through over-replication (carries additional copies of Octomom region) [37]
GCF_016584375.1	wMelOctoless	Complete Genome	wMelCS	Causes early death of the host through over-replication (the Octoless region is absent) [37]
GCF_016584405.1	wMelCS_b	Complete Genome	wMelCS	[37]
GCF_016584425.1	wMel	Complete Genome	wMel	[37]
GCF_017916155.1	FFD25	Scaffold	wMelCS	[75]
GCF_021347805.1	wMel_Trop	Scaffold	wMel	Sampled from a tropical climate [24]
GCF_021347845.1	wMel_Temp	Scaffold	wMel	Sampled from a temperate climate [24]

## Data Availability

Data have been deposited in the EUROPEAN ENA database (Project: PRJEB57437); the accession numbers are ERS14237169 for wMelPlus and ERS14237170 for wMelCS112 samples with Illumina MiSeq reads ERR10508789 and ERR10556501 for wMelPlus and wMelCS112, correspondingly, and MinION reads ERR10558084 and ERR10558085 for wMelPlus and wMelCS112, correspondingly. The annotated genome assemblies are available under accessions GCA_947533255 and GCA_947538885 for wMelPlus and wMelCS112, respectively.

## Supplementary Materials

The following supporting information can be downloaded at https://www.mdpi.com/article/10.3390/ijms232416212/s1.
