# Supplementary material for: New Wolbachia pipientis Genotype Increasing Heat Stress Resistance of Drosophila melanogaster Host Is Characterized by a Large Chromosomal Inversion
Source: Int J Mol Sci. 2022 Dec 19;23(24):16212. doi: 10.3390/ijms232416212 (PMC9786649; doi:10.3390/ijms232416212)
Supplement: Supplementary file 1 [file ijms-23-16212-s001.zip › ijms-2058940-supplementary/Supplement Tables S1-S3.pdf]

Table S1. The genes of wMelPlus *W. pipientis* strain located in the identified inversion. 'Hypothetical proteins' are excluded.

**locus_tag**	**length_bp**	**gene**	**EC_number**	**COG**	**product**
WMELPLUS_00339	363			COG3039	IS5 family transposase ISWpi1
WMELPLUS_00342	1173	sucB	2.3.1.61	COG0508	Dihydrolipoyllysine-residue succinyltransferase component of 2-oxoglutarate dehydrogenase complex
WMELPLUS_00343	909	hemC	2.5.1.61	COG0181	Porphobilinogen deaminase
WMELPLUS_00344	888	murB	1.3.1.98	COG0812	UDP-N-acetylenolpyruvoylglucosamine reductase
WMELPLUS_00351	1707			COG1132	putative ABC transporter ATP-binding protein
WMELPLUS_00353	1707	argS	6.1.1.19		Arginine--tRNA ligase
WMELPLUS_00358	624	ccmA	7.6.2.5	COG4133	Cytochrome c biogenesis ATP-binding export protein CcmA
WMELPLUS_00360	1866	aspS	6.1.1.12		Aspartate--tRNA ligase
WMELPLUS_00361	1263	proP_2			Proline/betaine transporter
WMELPLUS_00362	924	rluC	5.4.99.24	COG0564	Ribosomal large subunit pseudouridine synthase C
WMELPLUS_00363	981	acoA	1.1.1.-	COG1071	Acetoin:2,6-dichlorophenolindophenol oxidoreductase subunit alpha
WMELPLUS_00365	1320	miaB	2.8.4.3	COG0621	tRNA-2-methylthio-N(6)-dimethylallyladenosine synthase
WMELPLUS_00366	993	dtpA		COG3104	Dipeptide and tripeptide permease A
WMELPLUS_00367	522	dtpC		COG3104	Dipeptide and tripeptide permease C
WMELPLUS_00368	3336	ileS	6.1.1.5	COG0060	Isoleucine--tRNA ligase

WMELPLUS _00370	2340	pheT	6.1.1.20	COG0 072	Phenylalanine--tRNA ligase beta subunit
WMELPLUS _00372	726	atpB		COG0 356	ATP synthase subunit a
WMELPLUS _00373	228	atpH _1			ATP synthase subunit c
WMELPLUS _00374	480	atpG _1			ATP synthase subunit b'
WMELPLUS _00375	477	atpF			ATP synthase subunit b
WMELPLUS _00377	1053	ctaA	1.3.-.-	COG1 612	Heme A synthase
WMELPLUS _00378	2076	accA 1		COG4 770	Acetyl-/propionyl-coenzyme A carboxylase alpha chain
WMELPLUS _00380	1422	gltX1 _1	6.1.1.17		Glutamate--tRNA ligase 1
WMELPLUS _00382	1800	sdhA	1.3.5.1		Succinate dehydrogenase flavoprotein subunit
WMELPLUS _00384	618	gmk	2.7.4.8	COG0 194	Guanylate kinase
WMELPLUS _00387	1095	ychF		COG0 012	Ribosome-binding ATPase YchF
WMELPLUS _00389	1176	cca	2.7.7.72		CCA-adding enzyme
WMELPLUS _00393	1098	gap	1.2.1.12	COG0 057	Glyceraldehyde-3-phosphate dehydrogenase
WMELPLUS _00394	762	tatC		COG0 805	Sec-independent protein translocase protein TatC
WMELPLUS _00395	456	argR		COG1 438	Arginine repressor
WMELPLUS _00396	735	artP		COG0 834	Arginine-binding extracellular protein ArtP
WMELPLUS _00397	648	artQ		COG0 765	Arginine transport system permease protein ArtQ
WMELPLUS _00398	642	artM		COG1 126	Arginine transport ATP-binding protein ArtM
WMELPLUS _00399	363			COG3 039	IS5 family transposase ISWpi1
WMELPLUS _00400	342			COG3 039	IS5 family transposase ISWpi1
WMELPLUS _00406	675	pyrF	4.1.1.23	COG0 284	Orotidine 5'-phosphate decarboxylase
WMELPLUS _00407	1323	smc_ 2			Chromosome partition protein Smc

WMELPLUS _00409	1215	ftsH_ 1	3.4.24.-		ATP-dependent zinc metalloprotease FtsH
WMELPLUS _00410	2595	valS	6.1.1.9		Valine--tRNA ligase
WMELPLUS _00411	1110	smc_ 3			Chromosome partition protein Smc
WMELPLUS _00412	516	yrdA		COG0 663	Protein YrdA
WMELPLUS _00414	1590	pyrG	6.3.4.2	COG0 504	CTP synthase
WMELPLUS _00415	408	tadA	3.5.4.33	COG0 590	tRNA-specific adenosine deaminase
WMELPLUS _00416	1203	proP _3			Proline/betaine transporter
WMELPLUS _00418	1101	ftsH_ 2	3.4.24.-		ATP-dependent zinc metalloprotease FtsH
WMELPLUS _00419	999	bfmB AB	1.2.4.4	COG0 022	2-oxoisovalerate dehydrogenase subunit beta
WMELPLUS _00422	1797	lepA	3.6.5.-	COG0 481	Elongation factor 4
WMELPLUS _00429	741			COG0 217	putative transcriptional regulatory protein
WMELPLUS _00430	708	bam D		COG4 105	Outer membrane protein assembly factor BamD
WMELPLUS _00432	534	pgsA	2.7.8.5	COG0 558	CDP-diacylglycerol--glycerol-3-phosphate 3-phosphatidyltransfer ase
WMELPLUS _00433	1323	tme	1.1.1.40	COG0 280	NADP-dependent malic enzyme
WMELPLUS _00435	219			COG0 271	putative protein RP812
WMELPLUS _00436	324	grxD		COG0 278	Glutaredoxin 4
WMELPLUS _00437	1395	fumC	4.2.1.2	COG0 114	Fumarate hydratase class II
WMELPLUS _00438	1023	cgtA	3.6.5.-		GTPase Obg/CgtA
WMELPLUS _00439	1275	eno	4.2.1.11		Enolase
WMELPLUS _00440	1497	mur C	6.3.2.8	COG0 773	UDP-N-acetylmuramate--L-alanine ligase
WMELPLUS _00441	675	rlpA	4.2.2.-		Endolytic peptidoglycan transglycosylase RlpA
WMELPLUS _00445	1227	mtaB	2.8.4.5	COG0 621	Threonylcarbamoyla denosine tRNA

					methylthiotransferase MtaB
WMELPLUS_00446	1470	gatA	6.3.5.7	COG0154	Glutamyl-tRNA(Gln) amidotransferase subunit A
WMELPLUS_00454	1797	mutL_1		COG0323	DNA mismatch repair protein MutL
WMELPLUS_00455	441	rnhA_2	3.1.26.4		Ribonuclease H
WMELPLUS_00472	507	coaD	2.7.7.3	COG0669	Phosphopantetheine adenylyltransferase
WMELPLUS_00475	696	ispU	2.5.1.31	COG0020	Ditrans,polycis-undecaprenyl-diphosphate synthase ((2E,6E)-farnesyl-diphosphate specific)
WMELPLUS_00478	558	frr		COG0233	Ribosome-recycling factor
WMELPLUS_00479	744	pyrH	2.7.4.22	COG0528	Uridylate kinase
WMELPLUS_00480	861	tsf		COG0264	Elongation factor Ts
WMELPLUS_00481	849	rpsB		COG0052	30S ribosomal protein S2
WMELPLUS_00483	372	yajC		COG1862	Sec translocon accessory complex subunit YajC
WMELPLUS_00484	1821	nodM	2.6.1.16		Glutamine--fructose-6-phosphate aminotransferase [isomerizing]
WMELPLUS_00491	1116	ftsW	2.4.1.129		putative peptidoglycan glycosyltransferase FtsW
WMELPLUS_00492	717	ubiE	2.1.1.163	COG2226	Ubiquinone/menaquinone biosynthesis C-methyltransferase UbiE
WMELPLUS_00493	864	lipA	2.8.1.8		Lipoyl synthase
WMELPLUS_00494	315	rpmB		COG0227	50S ribosomal protein L28
WMELPLUS_00496	201	iscX		COG2975	Protein IscX
WMELPLUS_00498	615	rpsD		COG0522	30S ribosomal protein S4
WMELPLUS_00499	2073	tktA	2.2.1.1	COG0021	Transketolase 1
WMELPLUS_00502	1164	mdtL			Multidrug resistance protein MdtL
WMELPLUS_00508	558	dcd	3.5.4.13	COG0717	dCTP deaminase

WMELPLUS_00509	1017	pstS			Phosphate-binding protein PstS
WMELPLUS_00512	1059	trkI		COG0168	Trk system potassium uptake protein TrkI
WMELPLUS_00513	1371			COG2239	Magnesium transporter MgtE
WMELPLUS_00514	1011	prfB		COG1186	Peptide chain release factor RF2
WMELPLUS_00519	759	pstB	7.3.2.1		Phosphate import ATP-binding protein PstB
WMELPLUS_00520	786	dapB	1.17.1.8	COG0289	4-hydroxy-tetrahydrodipicolinate reductase
WMELPLUS_00524	801	znuB		COG1108	High-affinity zinc uptake system membrane protein ZnuB
WMELPLUS_00526	867	ispE	2.7.1.148	COG1947	4-diphosphocytidyl-2-C-methyl-D-erythritol kinase
WMELPLUS_00527	3270	addA	3.6.4.12		ATP-dependent helicase/nuclease subunit A
WMELPLUS_00532	1452	dnaB	3.6.4.12	COG0305	Replicative DNA helicase
WMELPLUS_00534	1638	metG	6.1.1.10		Methionine--tRNA ligase
WMELPLUS_00536	1176	COQ3_2	2.1.1.222		Ubiquinone biosynthesis O-methyltransferase, mitochondrial
WMELPLUS_00538	1749	dnaG	2.7.7.-		DNA primase
WMELPLUS_00539	1083			COG3177	putative protein
WMELPLUS_00540	1035	aaeA			p-hydroxybenzoic acid efflux pump subunit AaeA
WMELPLUS_00547	831	yqfL	2.7.11.32	COG1806	Putative pyruvate, phosphate dikinase regulatory protein
WMELPLUS_00548	711	ccmC		COG0755	Heme exporter protein C
WMELPLUS_00552	1281	purA	6.3.4.4	COG0104	Adenylosuccinate synthetase
WMELPLUS_00554	297			COG3039	IS5 family transposase ISCaa8
WMELPLUS_00556	264	infA			Translation initiation factor IF-1
WMELPLUS_00557	597	yhdE	3.6.1.9	COG0424	dTTP/UTP pyrophosphatase

WMELPLUS_00559	1344	alsT_1		COG1115	Amino-acid carrier protein AlsT
WMELPLUS_00563	381			COG3039	IS5 family transposase ISWpi1

Table S2. Genes of wMelPlus *Wolbachia* strain located in "Blue" unique inversion block – region (308921..316275). EC_number – the enzyme commission number if applicable (only for enzymes). COG – cluster of orthologous groups of proteins.

**locus_tag**	**Length (bp)**	**gene**	**EC_number**	**COG**	**product**
WMELPLUS_00339	363			COG3039	IS5 family transposase ISWpi1
WMELPLUS_00340	453				hypothetical protein
WMELPLUS_00341	1959				hypothetical protein
WMELPLUS_00342	1173	sucB	2.3.1.61	COG0508	Dihydrolipoyllysine-residue succinyltransferase component of 2-oxoglutarate dehydrogenase complex
WMELPLUS_00343	909	hemC	2.5.1.61	COG0181	Porphobilinogen deaminase
WMELPLUS_00344	888	murB	1.3.1.98	COG0812	UDP-N-acetylenolpyruvoylglucosamine reductase
WMELPLUS_00345	810				hypothetical protein

Table S3. Genes of wMelPlus *Wolbachia* strain located in "Yellow" unique inversion block – region (463923..531364). EC_number – the enzyme commission number if applicable (only for enzymes). COG – cluster of orthologous groups of proteins.

**locus_tag**	**Length (bp)**	**gene**	**EC_number**	**COG**	**product**
WMELPLUS_00489	741				hypothetical protein
WMELPLUS_00490	390				hypothetical protein
WMELPLUS_00491	1116	ftsW	2.4.1.129		putative peptidoglycan glycosyltransferase FtsW
WMELPLUS_00492	717	ubiE	2.1.1.163	COG2226	Ubiquinone/menaquinone biosynthesis C-methyltransferase UbiE
WMELPLUS_00493	864	lipA	2.8.1.8		Lipoyl synthase
WMELPLUS_00494	315	rpmB		COG0227	50S ribosomal protein L28
WMELPLUS_00495	342				hypothetical protein
WMELPLUS_00496	201	iscX		COG2975	Protein IscX
WMELPLUS_00497	87				tRNA-Leu(taa)
WMELPLUS_00498	615	rpsD		COG0522	30S ribosomal protein S4
WMELPLUS_00499	2073	tktA	2.2.1.1	COG0021	Transketolase 1
WMELPLUS_00500	189				WsnRNA46
WMELPLUS_00501	1629				hypothetical protein
WMELPLUS_00502	1164	mdtL			Multidrug resistance protein MdtL
WMELPLUS_00503	138				WsnRNA46
WMELPLUS_00504	816				hypothetical protein
WMELPLUS_00505	3336				hypothetical protein
WMELPLUS_00506	570				hypothetical protein
WMELPLUS_00507	360				hypothetical protein
WMELPLUS_00508	558	dcd	3.5.4.13	COG0717	dCTP deaminase
WMELPLUS_00509	1017	pstS			Phosphate-binding protein PstS
WMELPLUS_00510	933				hypothetical protein
WMELPLUS_00511	432				hypothetical protein
WMELPLUS_00512	1059	trkI		COG0168	Trk system potassium uptake protein TrkI
WMELPLUS_00513	1371			COG2239	Magnesium transporter MgtE
WMELPLUS_00514	1011	prfB		COG1186	Peptide chain release factor RF2
WMELPLUS_00515	165				hypothetical protein
WMELPLUS_00516	162				hypothetical protein
WMELPLUS_00517	198				hypothetical protein
WMELPLUS_00518	303				hypothetical protein
WMELPLUS_00519	759	pstB	7.3.2.1		Phosphate import ATP-binding protein PstB
WMELPLUS_00520	786	dapB	1.17.1.8	COG0289	4-hydroxy-tetrahydrodipicolinate reductase
WMELPLUS_00521	1047				hypothetical protein
WMELPLUS_00522	993				hypothetical protein
WMELPLUS_00523	156				hypothetical protein

WMELPLUS_00524	801	znuB		COG1108	High-affinity zinc uptake system membrane protein ZnuB
WMELPLUS_00525	366				hypothetical protein
WMELPLUS_00526	867	ispE	2.7.1.148	COG1947	4-diphosphocytidyl-2-C-methyl-D-erythritol kinase
WMELPLUS_00527	3270	addA	3.6.4.12		ATP-dependent helicase/nuclease subunit A
WMELPLUS_00528	291				hypothetical protein
WMELPLUS_00529	675				hypothetical protein
WMELPLUS_00530	597				hypothetical protein
WMELPLUS_00531	84				tRNA-Leu(caa)
WMELPLUS_00532	1452	dnaB	3.6.4.12	COG0305	Replicative DNA helicase
WMELPLUS_00533	1185				hypothetical protein
WMELPLUS_00534	1638	metG	6.1.1.10		Methionine--tRNA ligase
WMELPLUS_00535	441				hypothetical protein
WMELPLUS_00536	1176	COQ3_2	2.1.1.222		Ubiquinone biosynthesis O-methyltransferase, mitochondrial
WMELPLUS_00537	146				WsnRNA46
WMELPLUS_00538	1749	dnaG	2.7.7.-		DNA primase
WMELPLUS_00539	1083			COG3177	putative protein
WMELPLUS_00540	1035	aaeA			p-hydroxybenzoic acid efflux pump subunit AaeA
WMELPLUS_00541	165				hypothetical protein
WMELPLUS_00542	162				hypothetical protein
WMELPLUS_00543	198				hypothetical protein
WMELPLUS_00544	303				hypothetical protein
WMELPLUS_00545	315				hypothetical protein
WMELPLUS_00546	129				hypothetical protein
WMELPLUS_00547	831	yqfL	2.7.11.32	COG1806	Putative pyruvate, phosphate dikinase regulatory protein
WMELPLUS_00548	711	ccmC		COG0755	Heme exporter protein C
WMELPLUS_00549	171				hypothetical protein
WMELPLUS_00550	183				hypothetical protein
WMELPLUS_00551	1596				hypothetical protein
WMELPLUS_00552	1281	purA	6.3.4.4	COG0104	Adenylosuccinate synthetase
WMELPLUS_00553	153				hypothetical protein
WMELPLUS_00554	297			COG3039	IS5 family transposase ISCaa8
WMELPLUS_00555	2478				hypothetical protein
WMELPLUS_00556	264	infA			Translation initiation factor IF-1
WMELPLUS_00557	597	yhdE	3.6.1.9	COG0424	dTTP/UTP pyrophosphatase
WMELPLUS_00558	2679				hypothetical protein
WMELPLUS_00559	1344	alsT_1		COG1115	Amino-acid carrier protein AlsT
WMELPLUS_00560	330				hypothetical protein
WMELPLUS_00561	201				hypothetical protein
WMELPLUS_00562	408				hypothetical protein
WMELPLUS_00563	381			COG3039	IS5 family transposase ISWpi1
